# ProSave: an application for restoring quantitative data to manipulated subsets of protein lists

**DOI:** 10.1186/s13029-018-0070-0

**Published:** 2018-11-12

**Authors:** Daniel A. Machlab, Gabriel Velez, Alexander G. Bassuk, Vinit B. Mahajan

**Affiliations:** 1 Omics Laboratory, Stanford University, Palo Alto, CA USA; 2 Department of Ophthalmology, Byers Eye Institute, Stanford University, 1651 Page Mill Road, Palo Alto, CA 94304 USA; 3 Medical Scientist Training Program, University of Iowa, Iowa City, IA USA; 4 Department of Pediatrics, University of Iowa, Iowa City, IA USA; 5 Palo Alto Veterans Administration, Palo Alto, CA USA

**Keywords:** ProSave, Proteomics, Java, Precision medicine

## Abstract

**Background:**

In proteomics studies, liquid chromatography tandem mass spectrometry data (LC-MS/MS) is quantified by spectral counts or by some measure of ion abundance. Downstream comparative analysis of protein content (e.g. Venn diagrams and network analysis) typically does not include this quantitative data and critical information is often lost. To avoid loss of spectral count data in comparative proteomic analyses, it is critical to implement a tool that can rapidly retrieve this information.

**Results:**

We developed ProSave, a free and user-friendly Java-based program that retrieves spectral count data from a curated list of proteins in a large proteomics dataset. ProSave allows for the management of LC-MS/MS datasets and rapidly retrieves spectral count information for a desired list of proteins.

**Conclusions:**

ProSave is open source and freely available at https://github.com/MahajanLab/ProSave. The user manual, implementation notes, and description of methodology and examples are available on the site.

## Background

**Fig. 1:**
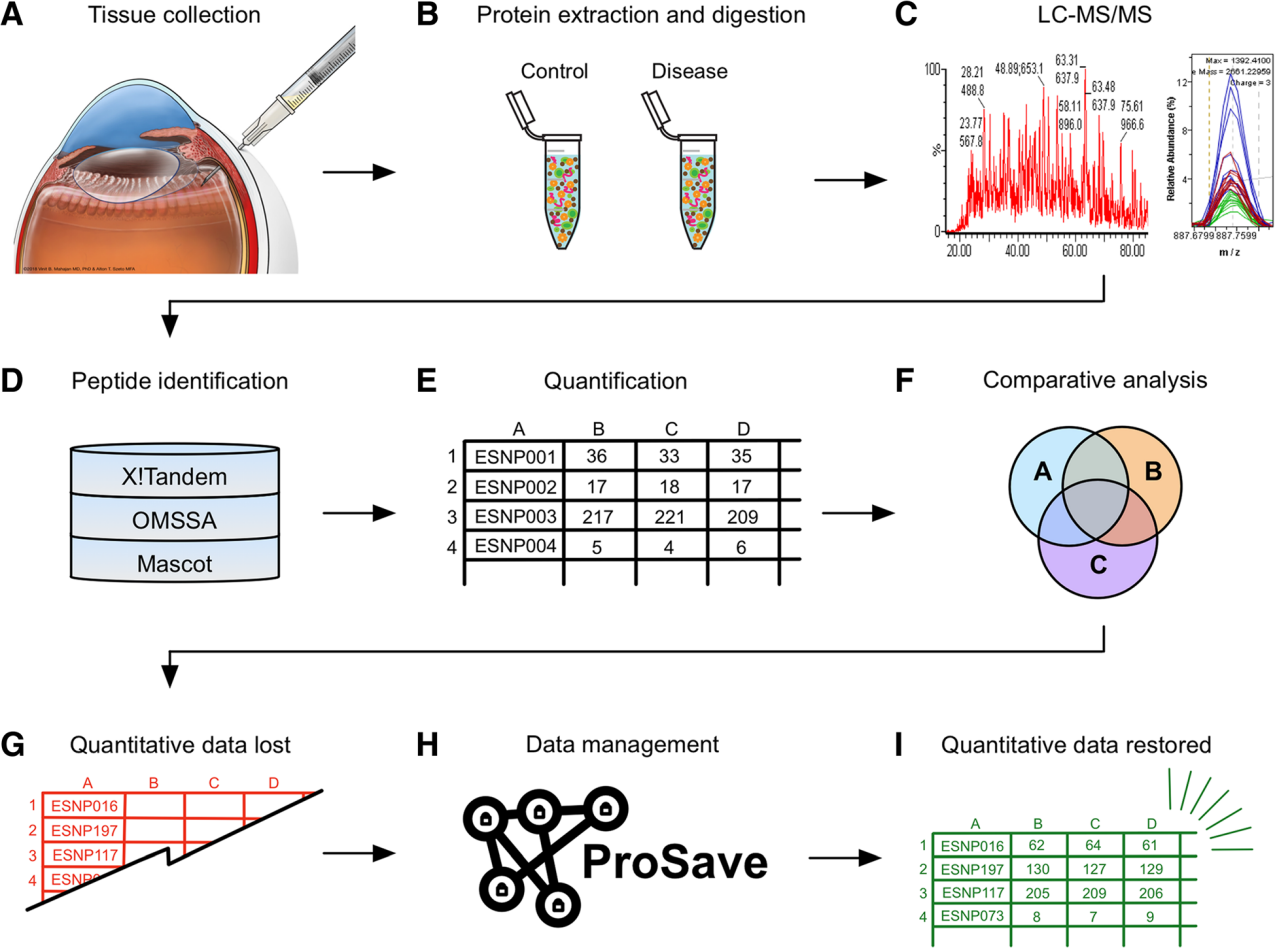
Informatics workflow for shotgun proteomics studies: **a** Liquid biopsies taken at time of surgery. **b** Liquid biopsies are processed for proteomic analysis. **c** Liquid chromatography-tandem mass-spectrometry used to analyze protein content. **d** Protein IDs are matched to peptide mass-spectral data. **e** Protein IDs and mass-spectra data are organized. **f** Samples (control vs. disease, etc.) are compared based on protein contents. **g** Quantitative data is lost during comparative analysis. **h** ProSave inputs original data and bare protein IDs, then outputs (**i**) restored protein-data pairs for trend analysis

Shotgun proteomic analysis is frequently used in translational biomedical research [1–5]. Mass spectrometry-based experiments generate large amounts of data, and the complexity and volume of this data is increasing with time. One promising application of shotgun proteomics is the molecular characterization of diseased tissue samples to identify biomarkers or drug targets [6]. We have applied this method to numerous vitreoretinal diseases where there are few therapeutic options [7, 8]. Liquid biopsies (e.g. vitreous or aqueous humor) can be taken at the time of surgery (Fig. 1a) [8–10]. These liquid biopsies can then be processed and analyzed using liquid chromatography-tandem mass spectrometry (LC-MS/MS) to evaluate protein content (Fig. 1b–c) [11]. Highly-advanced algorithms can match protein IDs to the thousands of peptide mass-spectral data obtained during the experiment (Fig. 1d) [12–15]. This quantitative data is typically represented in terms of spectral counts or ion abundance (Fig. 1e). Downstream analysis, organization, and meaningful interpretation of this LC-MS/MS data remains a challenge for researchers. Identified proteins can be further categorized using Venn diagrams, gene ontology (GO) categorization, clustering analysis, molecular pathway representation, and protein interaction network analysis (Fig. 1f) [1, 16, 17]. However, these analyses frequently make use of only the protein ID lists and the quantitative data (e.g. label-free spectral counts) is often ignored (Fig. 1g). This can create issues for investigators attempting to make meaningful interpretations of these results, especially if they are unfamiliar with shell scripting or lack access to expensive bioinformatics suites (e.g. Ingenuity or Partek). To overcome this barrier, we created ProSave, a Java-based application that restores quantitative data to manipulated lists of protein IDs from larger shotgun proteomics datasets (Fig. 1h–i). ProSave is different from other currently-available bioinformatic tools: it is free, open-source, and user-friendly (as opposed to R/Bioconductor).

## Implementation

ProSave was developed using Java and was successfully tested on Microsoft Windows 10 and Mac OS Sierra ver.10.12.6. It was written to maintain quantitative protein data (e.g. spectral counts, protein intensity, etc.) that was otherwise lost when protein ID lists were compared between tissue samples during proteomic analysis, which excludes all numerical protein data and focuses solely on the protein IDs derived from the liquid biopsies. ProSave solves this problem and restores critical protein information lost during analysis by processing original protein data before it is manipulated by downstream comparative analysis, such as Venn diagrams or gene ontology (GO) and network analysis. ProSave is a tool that is useful beyond proteomics research. It was designed to work with any large-scale gene or protein expression analysis. Further, ProSave works with protein expression data from a variety of methods, including data obtained through data-dependent and data-independent acquisition (DDA and DIA) as well as labeled methods like iTRAQ (isobaric tag for relative and absolute quantification) and SILAC (stable isotope labeling with amino acids in cell culture).

### Developer documentation

**Fig. 2:**
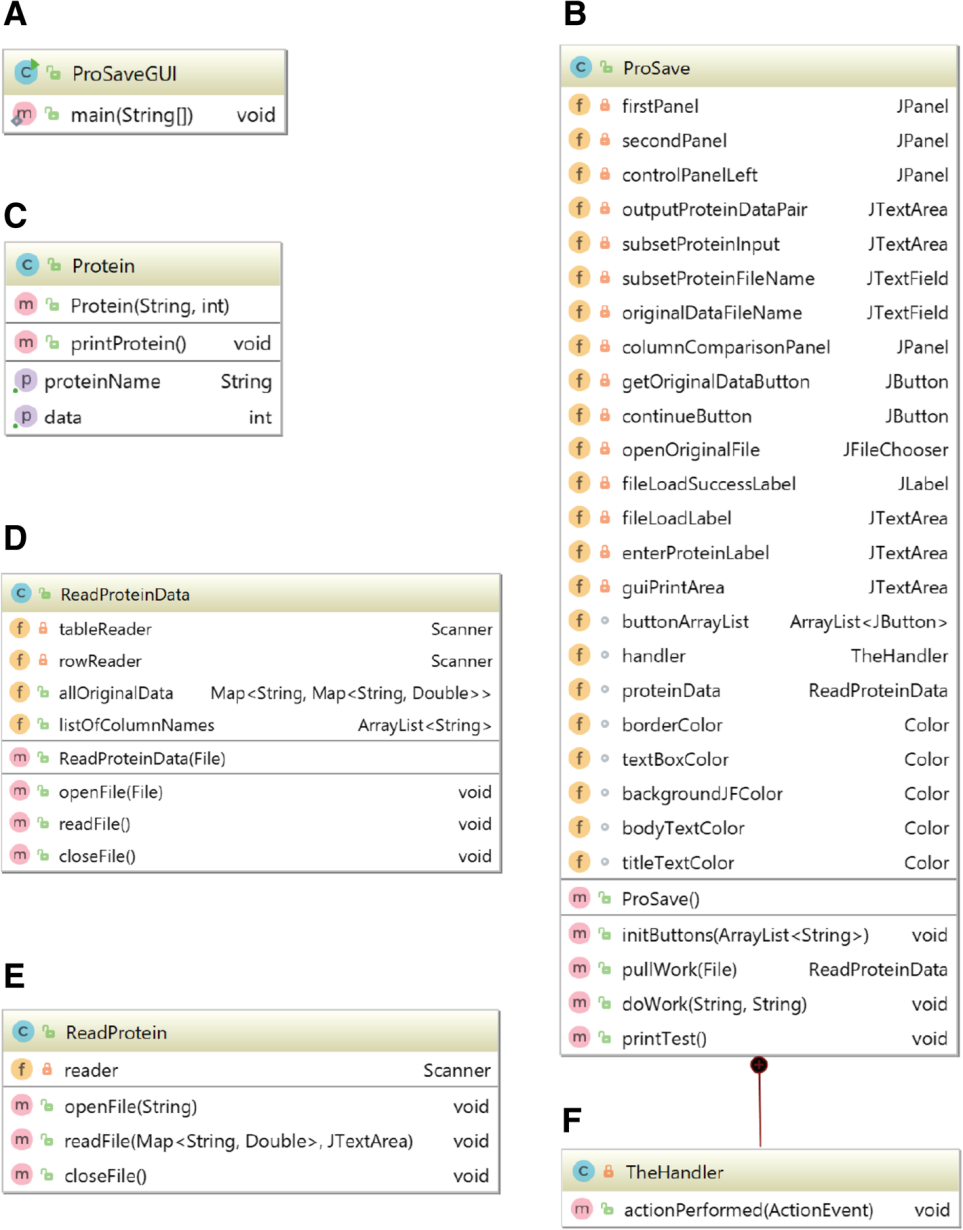
ProSave Java Class Diagram: **a** *ProSaveGUI* class creates the ProSave object and sets some GUI parameters. **b** The *ProSave* class creates the framework and manages layout of the GUI. **c** The *Protein* class stores data for a specific protein. **d** *ReadProteinData* organizes and stores original data from the file input. **e** The *ReadProtein* class organizes input proteins and retrieves data paired with each protein. **f** *TheHandler* manages actions of programs in response to user events on GUI

ProSave is a free, open source software available at https://github.com/MahajanLab/ProSave/. Additionally, java class files can be extracted from the ProSave.jar file for modification. The ProSaveGUI class creates the ProSave object and sets some graphical user interface (GUI) parameters (Fig. 2a). The *ProSave* class creates the framework and manages layout of the GUI (Fig. 2b). The *Protein* class is used to handle different types or amounts of data relating to each individual protein (Fig. 2c). The program processes the original data file by inserting data into a nested HashMap structure, executed by the *ReadProteinData* class (Fig. 2d). The *ReadProtein* class (Fig. 2e) uses the hashing structure for rapid data lookup. All GUI layout and interface parameters are specified in the *ProSave* class (Fig. 2b), which also has an internal class for event handling (Fig. 2f).

### User documentation

**Fig. 3:**
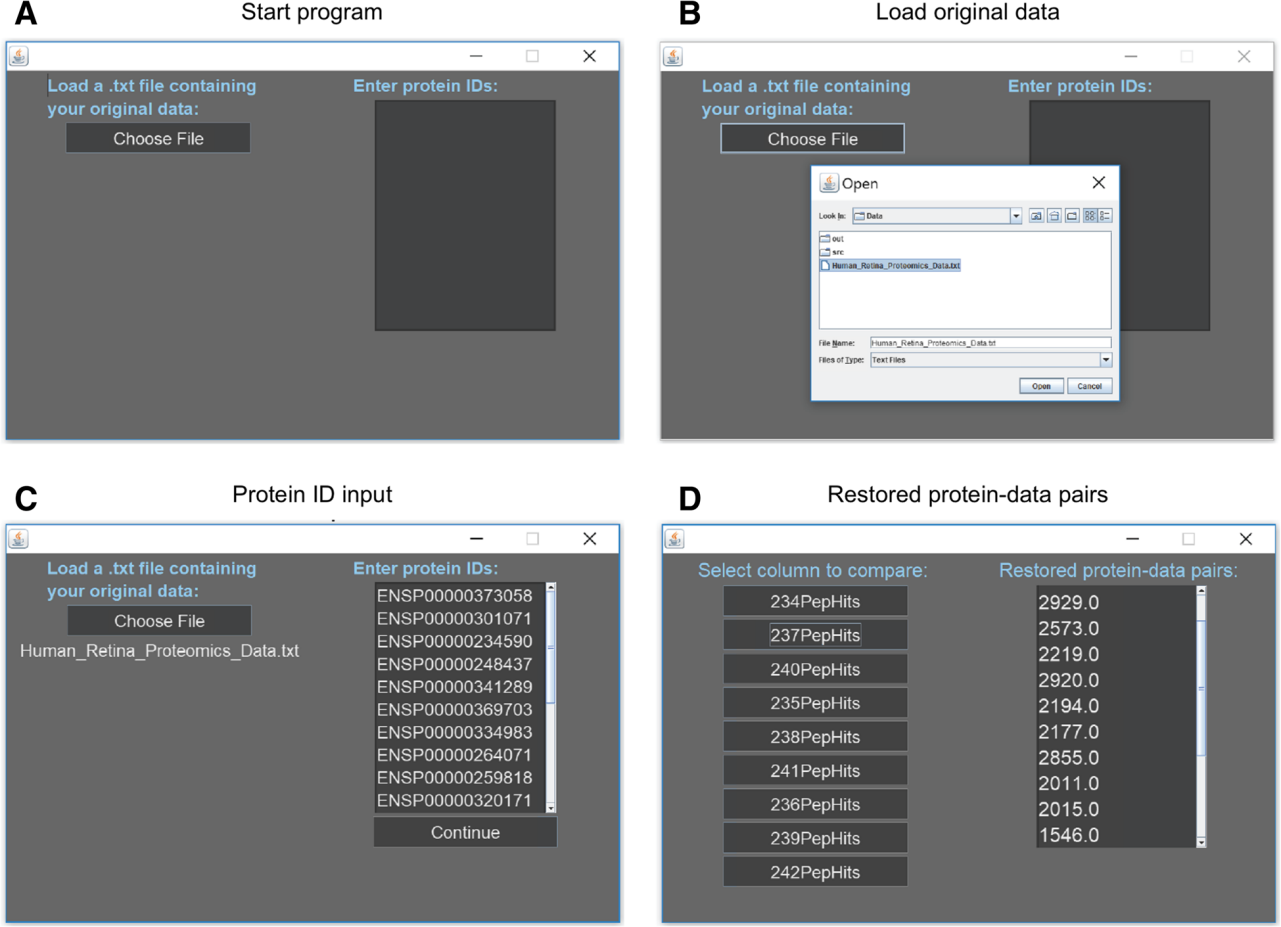
User documentation: **a** ProSave upon starting program. **b** Load original data by clicking ‘Choose File’ and selecting the file by browsing the file explorer. **c** Input of proteins which need data restored. **d** On left, tissues for comparison from original data, and on right, restored protein data from specified tissue in order of protein ID input

ProSave has been designed to be applied as a tool for any large-scale gene or protein expression investigation. Below are steps on how to use ProSave on any compatible data set:

- **Step 1:** Download ProSave.jar from https://github.com/MahajanLab/ProSave/ and run ProSave by opening the downloaded file (Fig. 3a). Additionally, download Java if it is not already downloaded.
- **Step 2:** Make a .txt with the original data. To do this from Excel go to File>Export>Change File Type>Text>Save. Once ProSave opens, click ‘Choose File’ to add the .txt file of the original data. For proper function, insure all columns have one-word names and text begins on first row of the .txt file (Fig. 3b).
- **Step 3:** Enter a list of protein IDs in the textbox labeled ‘Enter protein IDs’, then click ‘Continue’ (Fig. 3c).
- **Step 4:** Click the button labels with the name of the column of data corresponding to the tissue for comparison.
- **Step 5:** Get restored data from the text box labeled ‘Restored protein-data pairs’ (Fig. 3d).

## Results

### Case study

We tested ProSave on a comparative proteomics dataset of anatomical regions of the human retina: the peripheral retina, juxta-macular, and foveomacular regions [18]. LC-MS/MS was performed on retinal punch biopsies using an LTQ Velos and data were acquired using the DDA acquisition method as previously described. [18, 19] We identified 1,779 ± 51 individual proteins in the peripheral retina, 1,999 ± 46 individual proteins juxta-macular region, and 1,974 ± 92 individual proteins in the foveomacular region. Data were organized and analyzed using comparative analyses (e.g. Venn diagrams, differential protein expression, pathway representation, etc.). Protein ID lists from each tissue sample were compared using Venn diagrams to identify shared and unique proteins among the different regions of the retina. This analysis identified 1,354 proteins shared among the three retinal regions. After this comparison, however, only protein IDs remained, and the protein expression levels were not available for interpretation. Using ProSave, spectral count data was restored to this list of 1,354 proteins and we were able to ascertain the most abundant proteins shared among the three groups: alpha- and gamma-enolase, tubulin, pyruvate kinase, creatine kinase b-type, vimentin, glyceraldehyde-3-phosphate dehydrogenase, and histone H2B (types 1-D and G) [18]. A similar approach was used to gather information on the most abundant proteins unique to each anatomical region [18].

Without protein abundance data, insights into significant similarities or differences in retinal tissue protein expression are ambiguous. To avoid such data loss, one could attempt the tedious and time-consuming task of interrogating the original dataset to restore quantitative data for each protein of interest. Instead, ProSave accomplishes the same task in a matter of seconds instead of hours or days. We applied ProSave to our shared and unique protein lists to restore spectral count data. This gave us insight into which proteins were most and least abundant, thus allowing us to increase our understanding of targeted tissues.

## Conclusions

In conclusion, ProSave is a free and user-friendly tool to restore quantitative data to manipulated subsets of protein IDs during analysis of proteomic data. It speeds up the workflow for proteomic bioinformatics and makes for meaningful interpretation of comparative data. We anticipate that ProSave will be a useful tool to simplify processing and analysis of translational proteomics data. Such a program could even be applied to other gene/protein expression platforms where comparative analyses make use of only gene/protein IDs (e.g. RNA-seq, microarrays, ELISA).

## Availability and requirements

**Project name:** ProSave

**Project home page:** https://github.com/MahajanLab/ProSave

**Operating system(s):** Platform independent

**Programming language:** Java

**Other requirements:** None

**License:** GNU

**Any restrictions to use by non-academics:** None

## Abbreviations

DDA: Data-dependent acquisition
DIA: Data-independent acquisition
GO: Gene ontology
GUI: Graphical user interface
iTRAQ: Isobaric tag for relative and absolute quantification
LC-MS/MS: Liquid chromatography-tandem mass spectrometry
SILAC: Stable isotope labeling with amino acids in cell culture
